# Impact of accessory gene regulator (*agr*) dysfunction on vancomycin pharmacodynamics among Canadian community and health-care associated methicillin-resistant *Staphylococcus aureus*

**DOI:** 10.1186/1476-0711-10-20

**Published:** 2011-05-20

**Authors:** Brian T Tsuji, Robert D MacLean, Linda D Dresser, Martin J McGavin, Andrew E Simor

**Affiliations:** 1 Laboratory Antimicrobial Pharmacodynamics, School of Pharmacy and Pharmaceutical Sciences, University at Buffalo, New York 14260, USA; 2 The New York State Center of Excellence in Bioinformatics & Life Sciences, University at Buffalo, New York 14203, USA; 3 Roswell Park Cancer Institute Departments of Medicine, Buffalo, NY, 14203, USA; 4 Leslie Dan Faculty of Pharmacy University of Toronto, Toronto, Ontario, M5A 2N4, Canada; 5 Faculty of Medicine, University of Toronto, Toronto, Ontario, M5A 2N4, Canada; 6 Sunnybrook Health Sciences Centre, Toronto, Ontario, M4N 3M5, Canada

## Abstract

**Background:**

The accessory gene regulator (*agr*) is a quorum sensing cluster of genes which control colonization and virulence in *Staphylococcus aureus*. We evaluated *agr *function in community- (CA) and healthcare-associated (HA) MRSA, to compare the pharmacodynamics and bactericidal activity of vancomycin against *agr *functional and dysfunctional HA-MRSA and CA-MRSA.

**Methods:**

40 clinical isolates of MRSA from the Canadian Nosocomial Infection Surveillance Program were evaluated for delta-haemolysin production, as a surrogate marker of *agr *function. Time kill experiments were performed for vancomycin at 0 to 64 times the MIC against an initial inoculum of 10^6 ^and 10^8 ^cfu/ml of *agr *functional and dysfunctional CA-MRSA and HA-MRSA and these data were fit to a hill-type pharmacodynamic model.

**Results:**

15% isolates were *agr *dysfunctional, which was higher among HA-MRSA (26.3%) versus CA-MRSA (4.76%). Against a low initial inoculum of 10^6 ^cfu/ml of CA-MRSA, vancomycin pharmacodynamics were similar among *agr *functional and dysfunctional strains. However, against a high initial inoculum of 10^8 ^cfu/ml, killing activity was notably attenuated against *agr *dysfunctional CA-MRSA (USA400) and HA-MRSA (USA100). CA-MRSA displayed a 20.0 fold decrease in the maximal reduction in bacterial counts (Emax) which was 3.71 log_10 _CFU/ml for *agr *functional vs. 2.41 log_10 _CFU/ml for *agr *dysfunctional MRSA (p = 0.0007).

**Conclusions:**

Dysfunction in *agr *was less common among CA-MRSA vs. HA-MRSA. *agr *dysfunction demonstrated an impact on vancomycin bactericidal activity and pharmacodynamics against a high initial inoculum of CA-MRSA and HA-MRSA, which may have implications for optimal antimicrobial therapy against persistent, difficult to treat MRSA infections.

## Background

The accessory gene regulator *(agr) *is a quorum sensing cluster of genes which orchestrate the expression of cell-secreted and virulence factors, and several metabolic pathways in *Staphylococcus aureus *in a growth dependant fashion [1,2]. It has been hypothesized that *S. aureus *which exhibit *agr *dysfunction may possess an intrinsic survival advantage: these strains have demonstrated vancomycin tolerance and a proclivity to develop heterogeneous resistance under vancomycin selective pressure[3,4]. However, *agr *dysfunction has primarily been evaluated in strains of healthcare-associated (HA) MRSA where a down-regulated *agr *locus has been associated with prolonged and persistent bactaeremia [5,6].

Worldwide, the incidence of community-associated (CA) MRSA is rapidly increasing with the emergence of highly virulent strains in Canada [7-9]. While a large proportion of HA-MRSA display dysfunctional in the *agr *loci, the prevalence of *agr *dysfunction among CA-MRSA is relatively low from 3.5 to 9% [10,11]. Although this may account for the enhanced virulence of CA-MRSA as compared with HA-MRSA, whether dysfunction in *agr *would hamper vancomycin bactericidal activity among Canadian CA-MRSA has not been fully elucidated. Therefore, the objectives of this current study were to compare vancomycin pharmacodynamics of *agr *dysfunctional versus functional CA-MRSA and HA-MRSA clinical isolates from the Canadian Nosocomial Infection Surveillance Program at low and high initial inoculum.

## Methods

### Bacterial Isolates

40 clinical methicillin-resistant *S. aureus *(MRSA) isolates were selected for analysis obtained from the Canadian Nosocomial Infection Surveillance Program (CNISP), as shown in Table 1. Isolates were previously subtyped by using pulsed-field gel electrophoresis (PFGE) as previously described by Simor et.al. [8]. A community-associated isolate was defined as one which had a USA300/Canadian-MRSA-10 or USA400/Canadian-MRSA-7 PFGE profile, according to the nomenclature established by Simor et. al. and McDougal et. al. [8,12]. Isolates with other PFGE profiles were considered to be healthcare-associated. All isolates were evaluated for *agr *dysfunction using delta-haemolysin production as a surrogate marker of *agr *function using RN4220, as previously described [4]. Four strains subsequently were selected including two CA-MRSA (CA-MRSA 26, *agr *functional, USA300/Canadian-MRSA-10 and CA-MRSA 20, *agr *dysfunctional, USA400/Candian-MRSA-7) and two HA-MRSA (HA-MRSA 7, *agr *functional, USA100/Canadian-MRSA-2 and HA-MRSA 9, *agr *dysfunctional, USA100/Canadian-MRSA-2) for vancomycin time killing experiments. The PFGE profile of all isolates were blinded to the investigators performing time killing experiments (R.M. and B.T.) until final analyses were completed.

**Table 1:** Bacterial isolates utilized in this study

Community or Healthcare Associated	PFGE Profile (USA)	PFGE Profile (CANADA)
CA-MRSA (n = 21)*	USA300 (n = 15)	CMRSA-10 (n = 15)
	USA400 (n = 6)	CMRSA-7 (n = 6)
HA-MRSA (n = 19)**	USA100 (n = 8)	CMRSA-2 (n = 8)
	USA500 (n = 3)	CMRSA-5 (n = 3)
	USA600 (n = 6)	CMRSA-1 (n = 6)
Other	No USA Correlate (n = 2)	CMRSA-9 (n = 2)

*No CA-MRSA were *agr *dysfunctional

**6 HA-MRSA were *agr *dysfunctional,belonging to USA 100 (n = 1), USA 400 (n = 1), and USA 500 (n = 2), and USA 600(n = 2) PFGE profile. The remaining strains were classified as agr functional

### Antimicrobial, Media, and MIC Determination

Vancomycin (obtained from Sigma Chemical Company, St. Louis, Missouri) stock solutions were freshly prepared prior to each experimental run for all experiments. Minimum inhibitory concentrations were determined for each isolate in duplicate by microdilution techniques according to the guidelines of the Clinical and Laboratory Standards Institute (CLSI). Brain Heart Infusion broth (Difco, Detroit, MI) was utilized for time-kill experiments. Quantification of colony counts were determined using Tryptic Soy Agar II plates with 5% sheep Blood (BD, Franklin Lakes, NJ ).

### Time Kill Experiments

Selective time-kill experiments were performed in duplicate for vancomycin against *agr *functional and dysfunctional CA-MRSA and HA-MRSA using methodology as previous described[13]. Briefly, fresh bacterial colonies from an overnight growth were added to normal saline and adjusted spectrophotometrically to provide a standard suspension. This suspension was diluted with BHI and antibiotic stock solutions to achieve a starting inoculum of approximately 10^6 ^or 10^8 ^CFU/ml. Vancomycin concentrations of 0, 0.25, 1, 4, 16, and 64 times the MIC were tested against all isolates. Each 10-ml culture was incubated in a water bath at 35°C with constant shaking, and 0.1-ml samples were withdrawn for the determination of bacterial counts at 0, 4,8 and 24 h. Colony counts were determined by plating 50 μl of each diluted sample onto BHI agar (Becton Dickinson, Franklin Lakes, NJ) with an automated spiral dispenser (WASP; Don Whitley Scientific Limited, West Yorkshire, England) and incubating the plates for 24 h at 35°C to confirm colony counts.

### Pharmacokinetic and Pharmacodynamic Analyses

For each regimen tested, pharmcodynamic analysis was performed as previously described[13] using the Log Ratio Change comparing the changes in cfu/ml from 0 (CFU_0 h_) versus 24 h (CFU_24 h_) and calculated as shown in equation 1.(1)

Using non-linear regression a four-parameter concentration-effect Hill-type model was fit to the effect parameter Systat (Version 11, Richmond, VA) using:(2)

Equation 2, a sigmoidal maximal effect concentration effect relationship, was utilized to characterize the relationship between the decrease in bacterial counts from 0 to 24 h as characterized by the Log Ratio Change. The dependent variable (E, Effect) was the Log Ratio Change measuring the reduction in bacterial counts for each regimen comparing 0 h to 24 h, E_0 _is the measured effect at zero drug concentration which is an assessment of bacterial growth with no vancomycin, E_max _is the maximal effect which describes the maximal reduction of bacterial counts in log_10 _CFU/ml , C:MIC is concentration of vancomycin divided by its respective MIC, EC_50 _is the effective concentration of vancomycin for which there is 50% maximal effect, H is the slope constant also described as the Hill or sigmoidicity constant. Statistical comparisons among each model fitted parameter were performed using the t-test (Systat 11, Richmond, VA), for which a p value of <0.05 was considered significant.

## Results

**Figure 1:**
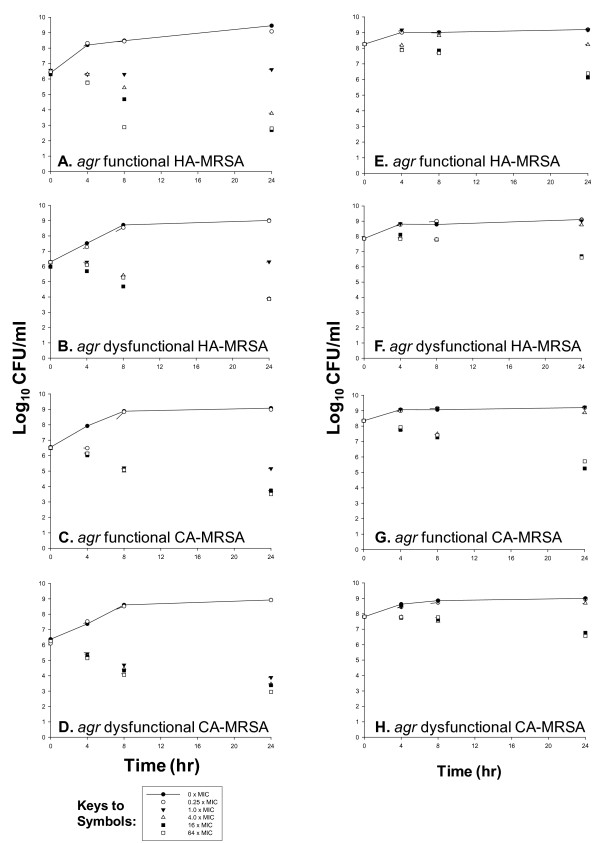
**Time kill experiments evaluating the bactericidal activity of vancomycin versus *agr *functional HA-MRSA 7 (A,E), *agr *dysfunctional HA-MRSA 9 (B,F), *agr *functional CA-MRSA 26 (C,G), and *agr *dysfunctional CA-MRSA 20 (D,H) at low and high initial inoculum (10^6^, 10^8 ^log_10 _cfu/ml)**.

Vancomycin MICs against HA-MRSA 7, 9, were 1.0, 2.0 mg/L and for CA-MRSA 26 and 20 were 1.0, and 1.0 mg/L. 15% (6 of 40) of isolates were dysfunctional in *agr *which was higher among HA-MRSA (26.3%, 5 of 19) vs. CA-MRSA (4.76%, 1 of 21, USA 400/Canadian MRSA 7). The activity of vancomycin against a low (10^6 ^cfu/ml) and high (10^8 ^cfu/ml) initial inoculum of bacterial strains is depicted in Figure 1. *agr *functional strains (CA-MRSA 26) displayed greater killing versus *agr *dysfunctional MRSA (CA-MRSA 20). Maximal bacterial reductions at 64 times the MIC for CA-MRSA 20 and CA-MRSA 26 were 3.30 and 3.01 log_10 _cfu/ml at 24 h, respectively. However, against 10^6 ^cfu/ml inoculum differences in vancomycin killing of HA-MRSA with respect to *agr *function were noted with less activity versus *agr *dysfunctional strains. Interestingly, against a high initial inoculum of MRSA, vancomycin did not achieve bactericidal activity against any isolate. Most notably, against 10^8 ^cfu/ml, the killing activity of vancomycin against *agr *functional versus dysfunctional strains consistently differed for both CA-MRSA and HA-MRSA strains. Greater vancomycin killing activity occurred against *agr *functional (HA-MRSA 7, CA-MRSA 26) versus dysfunctional (HA-MRSA 9, CA-MRSA 20) strains. Reductions in bacterial counts at 24 h were greater against HA-MRSA 7 versus HA-MRSA 9 with 1.87 versus 1.24 log_10 _CFU/ml reduction at 24 h. Against CA-MRSA 20, killing approached the bactericidal threshold with a 2.63 log_10 _cfu/ml reduction as compared with CA-MRSA 26 where a decrease of only 1.25 log_10 _cfu/ml at 24 h was demonstrated.

**Figure 2:**
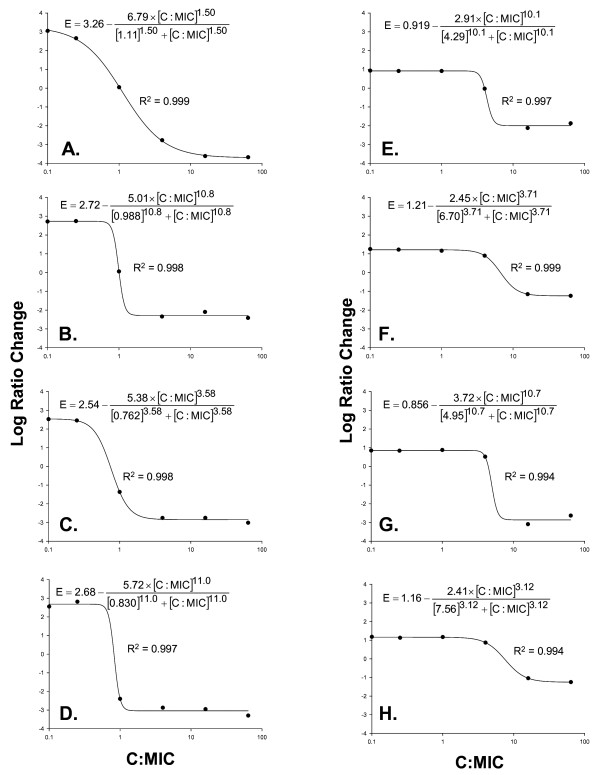
**Pharmacodynamic relationship evaluating vancomycin concentration to minimum inhibitory concentration ratio (C:MIC) and change in log10 CFU/ml at 24 hours (Log Ratio Change) among *agr *functional HA-MRSA 7 (A,E), *agr *dysfunctional HA-MRSA 9 (B,F), *agr *functional CA-MRSA 26 (C,G), and *agr *dysfunctional CA-MRSA 20 (D,H) at low and high initial inoculum (10^6^, 10^8 ^log_10 _cfu/ml)**. R^2^: Coefficient of Determination. Data are reported as maximum likelihood model fitted parameter estimates from equation 2 for each strain.

Analysis of pharmacodynamics (PD) revealed excellent model fits of the data to the Hill model. R^2 ^for was >0.99 for all MRSA isolates at both initial inoculum. The concentration effect profile is depicted in Figure 2 with maximal likelihood fitted parameters from equation 2. Reduction in bacterial counts with vancomycin occurred in a concentration-dependent manner, as seen with the steep sigmoidicity constant (H) and low EC50 against most isolates. All pharmacodynamic parameter estimates consistently differed for both HA-MRSA and CA-MRSA when comparing *agr *functional versus dysfunctional isolates at the higher starting inoculum (10^8^). There were significant differences in regards to vancomycin activity in reducing bacterial counts when comparing *agr *function and dysfunctional strains among HA-MRSA and CA-MRSA. HA-MRSA isolates displayed a 2.89 fold decrease in the maximal reduction in bacterial counts (Emax) which was 2.91 log_10 _CFU/ml for *agr *functional vs. 2.45 log_10 _CFU/ml for *agr *dysfunctional MRSA log_10 _CFU/ml (p = 0.0163).

CA-MRSA displayed an even greater trend with a 20.0 fold decrease in the maximal reduction in bacterial counts (Emax) which was 3.71 log_10 _CFU/ml for *agr *functional vs. 2.41 log_10 _CFU/ml for *agr *dysfunctional MRSA (p = 0.0007). The sigmoidicity constant (H) was nearly three times higher and EC_50 _values were consistently lower in *agr *functional versus dysfunctional isolates, but did not achieve significance. Model fitted for each isolate parameter estimates from Equation 2 in Figure 2.

## Discussion

The epidemiology of *Staphylococcus aureus *is rapidly changing. Highly virulent strains of CA-MRSA have emerged worldwide and have been associated with fatal infections in a plethora of recent outbreaks including severe necrotizing pneumonia and fasciitis and in both children and healthy adults [8,9,14]. A number of virulence determinants have recently been characterized in CA-MRSA including alpha-type phenol soluble modules (PSMs), the arginine catabolic mobile element (ACME), the panton- valentine leucocidin cytotoxin and a variety of virulence factors not common in HA-MRSA[15-17]. Interestingly, many of these virulence determinants are regulated via the two component *agr *system, the master regulator of *S. aureus*, controlling growth, colonization and virulence[1,17,18]. However, although *agr *dysfunction in *S. aureus *may potentially decrease virulence, the loss of *agr *function may increase the proclivity to develop vancomycin tolerance and resistance, although this has not been previously evaluated in strains of CA-MRSA. Therefore, we sought to determine if dysfunctional *agr *strains in either HA-MRSA and CA-MRSA would impact vancocmycin pharmacodynamics.

In the current investigation, *agr *dysfunction resulted in attenuation of vancomycin killing activity with decreases in maximal effect and lack of bactericidal activity against both HA-MRSA and CA-MRSA. We also determined that strains which exhibited *agr *dysfunction were far less common among CA-MRSA vs. HA-MRSA, with only one out of 18 isolates demonstrating this phenomena. Taken together, these findings may have potential implications for the use of vancomycin for the treatment of CA-MRSA infections. First, although reports of persistent bacteraemia, vancomycin treatment failure and heterogeneous resistance have been primarily in HA-MRSA, the findings of the present study highlight similarities of CA-MRSA and HA-MRSA when exposed to vancomycin [5,6]. Similar to previous reports in HA-MRSA, limited bacterial killing was evident by vancomcyin against CA-MRSA, especially in the context of *agr *dysfunction. Second, with reports of CA-MRSA as an emerging pathogen in infections that are sequestered or deep-seated, such as endocarditis, the potential for attenuation of bactericidal activity should be considered when vancomycin is utilized against a high initial inoculum of CA-MRSA [19]. Therefore, the inoculum effect displayed by vancomycin displays may be a potential explanation for the lack of bactericidal activity shown in this study. This is especially important to consider since eradication of *S. aureus *from sites of infection that are at high bacterial density or in a biofilm matrix at may be particularly difficult for vancomycin due to its pharmacodynamics properties, including slow bactericidal activity and the heterogeneous nature of vancomycin resistance. Finally, as the antimicrobial susceptibility profile of CA-MRSA is commonly greater than HA-MRSA and with initial reports of heterogeneous vancomycin-intermediate resistance in CA-MRSA emerging[20], the treatment of *agr *dysfunctional CA-MRSA may favor the use of combination therapy or alternative agents rather than glycopeptide monotherapy.

Potential limitations of this study include the limited number of strains including only two CA-MRSA and two HA-MRSA isolates which were utilized to evaluate vancomycin efficacy. We also acknowledge the lack of inclusion of a USA300 strain in time kill experiments because there were no *agr *dysfunctional CA-MRSA strains among the 40 isolates which we screened. Interestingly, these data corroborate our previous work which also shows an exceptionally low prevalence (2.5%) of *agr *dysfunction in community isolates of MRSA. Therefore, further *in vitro *and *in vivo *studies are necessary to confirm these findings in additional CA-MRSA before these results are applied to clinical practice.

## Conclusions

This study is the first to demonstrate that vancomycin activity was impaired against a high inoculum of both community and healthcare associated strains of MRSA that were dysfunctional in *agr*. Additionally this study characterized the function on *agr *among MRSA strains which were genetically defined according to community and hospital original and determined that *agr *dysfunction were far less common among CA-MRSA as compared with HA-MRSA (11). These result are important to consider particularly in the treatment of infections of high bacterial density due to MRSA when vancomycin is utilized against a high initial inoculum of CA-MRSA

## Competing interests

The authors declare that they have no competing interests.

## Authors' contributions

BTT was responsible for overall study design, participated in the experimental work, conducted an extensive literature review, and wrote the manuscript. RCM carried out the experimental work and participated in study design. LDD participated in the study design and contributed to writing of the manuscript. MJM contributed ideas and characterization of strains. AES characterized bacterial isolates, and provided his experience in bacterial genetics, contributed to study design, was involved in the writing of the manuscript. All authors have read and approved the final manuscript.
